# *Thelazia callipaeda* as a potential new threat to european wildcats: insights from an eco-epidemiological study

**DOI:** 10.1007/s11259-023-10071-8

**Published:** 2023-01-17

**Authors:** Elena Bertos, Mariola Sánchez-Cerdá, Emilio Virgós, José M. Gil-Sánchez, Marcos Moleón

**Affiliations:** 1 Department of Zoology, University of Granada, Granada, Spain; 2 Harmusch - Asociación de Estudio y Conservación de Fauna, Almodóvar del Campo, Ciudad Real, Spain; 3 Department of Biology and Geology, Physics and Inorganic Chemistry, University Rey Juan Carlos, Móstoles, Madrid, Spain

**Keywords:** *Felis catus*, *Felis silvestris*, Mediterranean, Nematode, Non-native parasite spread

## Abstract

Global warming and displacement of vectors and wild and domestic hosts by humans greatly change host-parasite interactions and parasite transmission rates. *Thelazia callipaeda* Railliet and Henry, 1910 (Spirurida: Thelaziidae) is a zoonotic parasite rapidly colonizing Europe from its Asian native range. This nematode is vectored by *Phortica* flies and may cause ocular disorders, such as keratitis and corneal ulcers, in wild mammals, dogs, cats and humans. With the aim of detecting possible threats for the European wildcat (*Felis silvestris*) in one of its last Mediterranean strongholds, we initiated in 2017 a long-term monitoring program in Sierra Arana (southeastern Spain), which includes exploring its epidemiological and spatial relationships with domestic cats (*Felis catus*). During routine medical check-ups, we detected ocular nematodes in 3 of 17 (17.6%) wildcats and 1 of 23 (4.3%) domestic cats tested, confirmed to be *T. callipaeda* by microscopic and molecular analyses. This nematode species was first detected in the study area in 2021. To our knowledge, this is the first case of infection in wildcats in Spain. Through telemetry, we detected little spatial overlap between the home ranges of wildcats and domestic cats, which seems to be sufficient to facilitate the inter-specific transmission of *T. callipaeda*. Our findings suggest that this pathogen could be a new threat to the wildcat, a locally endangered wild felid. Further eco-epidemiological monitoring and sanitary control of domestic cats will be strongly needed, especially given the ongoing global warming scenario.

**Supplementary Information:**

The online version contains supplementary material available at 10.1007/s11259-023-10071-8.

## Introduction

Ecosystems are rapidly changing due to anthropogenic stressors, which may greatly modify host-parasite interactions and parasite transmission rates (Cable et al. 2017). For instance, intentional and unintentional displacement of vectors and wild and domestic hosts by humans have led to range expansions in many parasite species around the globe (Crowl et al. 2008). In addition, global warming may help to increase parasites’ distribution range and alter their life cycles (Okulewicz 2017; Pombi et al. 2020), as well as to colonize new host species (Caron et al. 2013).

This may be the case of the nematode *Thelazia callipaeda* Railliet and Henry, 1910 (Spirurida: Thelaziidae), a vector-borne zoonotic parasite affecting the dog (*Canis familiaris*), cat (*Felis catus*), wild carnivores, lagomorphs, and humans (Otranto et al. 2021). The range and prevalence of this nematode are increasing, possibly due to the overseas movement of pets from countries where *T. callipaeda* is endemic (Silva et al. 2020) and the increasingly favorable environmental conditions for its vector, *Phortica variegata*, which seems to prefer warm temperatures (González et al. 2022). *Phortica* flies (specifically, males) feed on ocular secretions of mammals, thus being exposed to the first larval stage of *T. callipaeda*; if infected, these flies act as vectors (Máca and Otranto 2014). Infected mammals show conjunctival hyperemia, follicles and/or papillae in tarsal connective tissue, keratitis, and even corneal ulcers in severe infections (Guitián-Deltell et al. 2019).

Native to Asia, *T. callipaeda* has colonized 19 European countries (Marino et al. 2020) since its detection in Italy in 1989 (Rossi and Bertaglia 1989). In Europe, besides pets and humans, *T. callipaeda*’s hosts include seven wild mammalian carnivore species (European wildcat *F. silvestris*, Eurasian badger *Meles meles*, stone marten *Martes foina*, red fox *Vulpes vulpes*, golden jackal *C. aureus*, gray wolf *C. lupus*, and brown bear *Ursus arctos*) and two lagomorphs (European rabbit *Oryctolagus cuniculus*, and brown hare *Lepus europaeus*; Otranto et al. 2009; Gama et al. 2016; Mihalca et al. 2016; Pavlović et al. 2017; Ionică et al. 2019; Diakou et al. 2021 Papadopoulos et al. 2021; Bezerra-Santos et al. 2022, Cotuţiu et al. 2022). In Spain, this parasite was first detected in 2010 in a dog in the central-western part of the country (Guisado and Sanz 2010). Since then, in this country, *T. callipaeda* has been found in domestic cats (Hernández et al. 2012; Marino et al. 2015), red foxes (Calero-Bernal et al. 2013; Fidalgo et al. 2017), gray wolves (Nájera et al. 2020), and humans (Fuentes et al. 2012; López Medrano et al. 2015; Guitián-Deltell et al. 2019).

The European wildcat is a medium-sized wild felid, listed as Least Concern in the IUCN Red List, although several pieces of evidence indicate that the Mediterranean populations of the Iberian Peninsula are seriously threatened (Gerngross et al. 2022). For instance, a recent large-scale field survey confirmed that the populations of southern Spain have very low density and are highly fragmented (Gil-Sánchez et al. 2020). To study the factors that rule the population dynamics of Mediterranean wildcats, in 2017, we initiated a long-term monitoring program in one of the southern strongholds of the Iberian population by using different techniques such as camera-trapping, telemetry monitoring, and medical check-ups. This program includes the domestic cat, as a potential pathogen-transmitting agent for wildcats. In addition, both felines may play an epidemiological role in the wildlife-domestic-human interface, for instance, in relation to *T. callipaeda* (Otranto et al. 2021). Thus, this monitoring program could reveal important eco-epidemiological insights.

Here, we aimed to report the results of the systematic survey of *T. callipaeda* in wildcats and sympatric domestic cats in southern Spain and to discuss these findings in relation to the spatial relationships between both species in the study area. This may help to understand the role of each feline species as a reservoir of this parasite. Doing this, to our knowledge, we report the first cases of infection of *T. callipaeda* in wildcats in Spain.

## Materials & methods

### Study area and field surveys

The study area is located in Sierra Arana (Granada, southeastern Spain), a mountainous area (altitude: 850–2027 m a.s.l.) characterized by dry Mediterranean climate (annual average temperature: 17–22ºC; annual average precipitation: 200–400 mm; Rivas-Martínez 1987). Vegetation is dominated by plantation and natural pine forests (dominated by *Pinus halepensis*), Mediterranean schrubland (mainly, *Quercus ilex, Juniperus oxycedrus, Rosmarinus officinalis*, and *Stippa tenacissima*), and cereal and olive crops. Besides agriculture, the main land uses in this unprotected area include livestock farming (mostly, sheep and goats) and both small and big game hunting. The local wildcat population occupies a patch of Mediterranean scrubland of about 100 km^2^, with an estimated density of 0.17 ind./km^2^, being one of the highest abundances detected in Southern Spain (Gil-Sánchez et al. 2020). Domestic cats are distributed in ten farms and within four villages, reaching abundances of up to 12 individuals/farm and several tens in the villages.

Since 2017, we have captured 17 wildcats. To avoid disturbances to the wildcat’s breeding season, captures occurred in October-February. We distributed trap cages (100 × 50 × 75 cm) in the study area, baited with live pigeons. Pigeons were provided with food and water and protected against predators and climatic conditions with shelter (see Martin-Díaz et al. 2018). Wildcats were moved to a nearby biological station using a mobile cage and then anesthetized with a combination of ketamine (3.75 mg/kg; Imalgene 100 mg/ml, Boehringer Ingelheim Animal Health, Spain), medetomidine (0.04 mg/kg; Domtor 1 mg/ml, CP Pharma Handelsges, Germany), and midazolam (0.25 mg/kg; Midazolam Normon 15 mg/3ml, Normon Laboratories S.A., Spain). Heart rate, respiratory rate and body temperature were registered at 5 min intervals for a total handling time of 35–40 min. After that, most wildcats were recovered with atipamezole (0.125 mg/kg; Revertor 5 mg/ml, CP-Pharma Handelsges, Germany). During handling, all wildcats underwent a complete routine health evaluation (including weighing, taking morphometric measurements, general external examination, and revision of the auricular, ocular and oral cavities) and were checked on the inner side of the third eyelids of both eyes as part of their medical check-up. The taxonomic identification of the captured wildcats was based on coat patterns, which is feasible in this area given the absence of hybridization with domestic cats confirmed through genetic analyses (see Ballesteros-Duperón et al. 2014). We also trapped and checked 23 domestic cats in the study area since 2018, mainly during spring, i.e., after the wildcat capturing campaigns. In this case, the cages were baited with commercial wet cat food. The handling protocol was the same as for wildcats. During the ocular examination of wildcats and domestic cats, all nematode parasites (eyeworms) observed in their conjunctival sacs (Guitián-Deltell et al. 2019) were collected using sterile cotton swabs, forceps, and flushing with physiological saline solution (NaCl 0.9%). The nematodes were stored in vials containing 70% ethanol for species identification (see next section). We also inspected for possible lesions in the eyes caused by the presence of worms, and classified them according to the three stages defined by Marino et al. (2021): (1) no ocular signs; (2) mild conjunctivitis, ocular discharge or blepharitis, and (3) complicated conjunctivitis, keratitis, chemosis and/or purulent ocular discharge. In addition, we used fluorescein stain to detect possible ocular ulcers presumably caused by the nematodes.

Since 2017, 12 out of the 17 captured wildcats have been fitted with VHF transmitters (Sirtrack® and Lotek®) weighing < 60 g, with a mortality sensor, and a battery life of approximately 2.5 years. The radio-tracking routine consisted of trying to obtain three locations per individual a week (Martin-Díaz et al. 2018). In 2021 and 2022, two domestic cats (adult males) from one farm were equipped with GPS collars (Tellus Micro®) weighing < 250 g. The schedule consisted of four locations per day (0:00, 4:00, 12:00, and 19:00) downloaded by a VHF transceiver.

### Morphological and molecular identification of nematodes

The collected nematodes were identified using standard morphological identification keys (Otranto et al. 2003a). Nematodes were temporarily mounted on slides and observed under a light microscope (10-40x). Photographs were taken to facilitate the identification of the characteristic, sex-specific anatomical structures of the nematodes. We also recorded the presence of eggs and larvae in the case of females.

The morphological examination was complemented by molecular analysis to confirm the species. We analyzed one nematode per individual host to accomplish this purpose. Nematodes were washed several times with sterile double distilled water and then macerated until entirely homogenized. The extraction of nucleic acids from the obtained suspension was performed with automatic extraction equipment using KingFisher magnetic particles (ThermoScientific) by means of MagMax CORE Nucleic Acid Purification Kit reagents (Applied Biosystems), following the manufacturer’s instructions. Amplification of a region of the cox1 gene was performed using 100 ng of DNA in a final reaction volume of 50 l. NZYTaq DNA Polymerase reagents (NZYTech) were used for this reaction using a single PCR assay with 40 cycles and a hybridisation temperature of 52ºC. The concentrations used in the reaction mixture were: 2 mM magnesium chloride, 0.5 mM dNTPs, and 0.5 µM primers. NTF (5′-TGATTGGTGGTTTTGGTAA-3′) and reverse NTR (5′-ATAAGTACGAGTATCAATATC-3′) primers were used for PCR analysis. Detection of the amplified mitochondrial cytochrome c oxidase subunit 1 (cox1) gene was performed by electrophoresis in a 3% agarose gel for 30 min and a voltage of 126 V.

### Data analyses

For a given species, the prevalence of *T. callipaeda* was calculated as the number of infected individuals divided by the total number of individuals checked. We considered that an individual was infected if we detected the nematode at least during one of the check-ups (see Table S1). During a given capturing campaign, every cat was sampled only once (but note that a given cat could have been re-sampled in different campaigns). The difference in the prevalence between wildcats and domestic cats was tested through a Chi^2^ test on a 2 × 2 table. To assess the inter-specific proximity as a proxy of the inter-specific transmission risk (the closer the home ranges, the higher the transmission risk), we visually compared the telemetry locations between wildcats and domestic cats. Geospatial representation of the telemetry data was run in QGIS software v.3.16.6 “Hannover” using ETRS89/UTM zone 30 N (EPSG:25,830) and an orthophoto obtained from the National Plan for Aerial Orthophotography (https://pnoa.ign.es/, accessed on 26 February 2021).

## Results

**Fig. 1:**
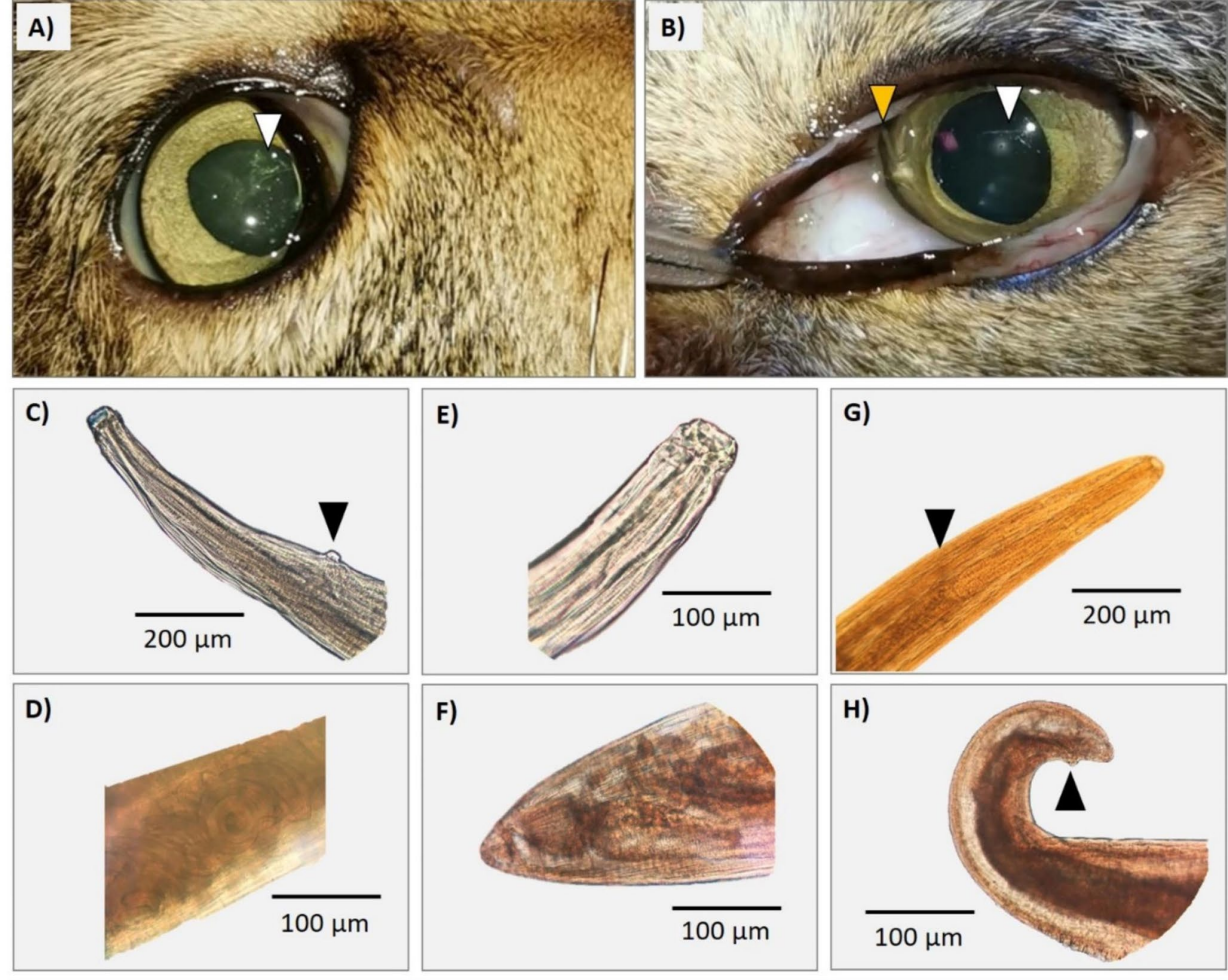
Images from affected wildcats and *Thelazia callipaeda* nematodes in Sierra Arana, southeastern Spain. Superficial ulcers in wildcat eyes after fluerescein test from H3 (A) and H5 (B) are shown by white triangles; in panel B), a nematode is indicated by an orange triangle. Microphotographs of *T. callipaeda* found in wildcats: female (C: anterior region of the body with the vulva; D: larvae inside the uterus; E: hexagonal buccal capsule; F: caudal area of the body) and male nematodes (G: anterior region of the body with the esophago-intestinal junction; H: curved tail with spicules). Black triangles show diagnostic features

**Fig. 2:**
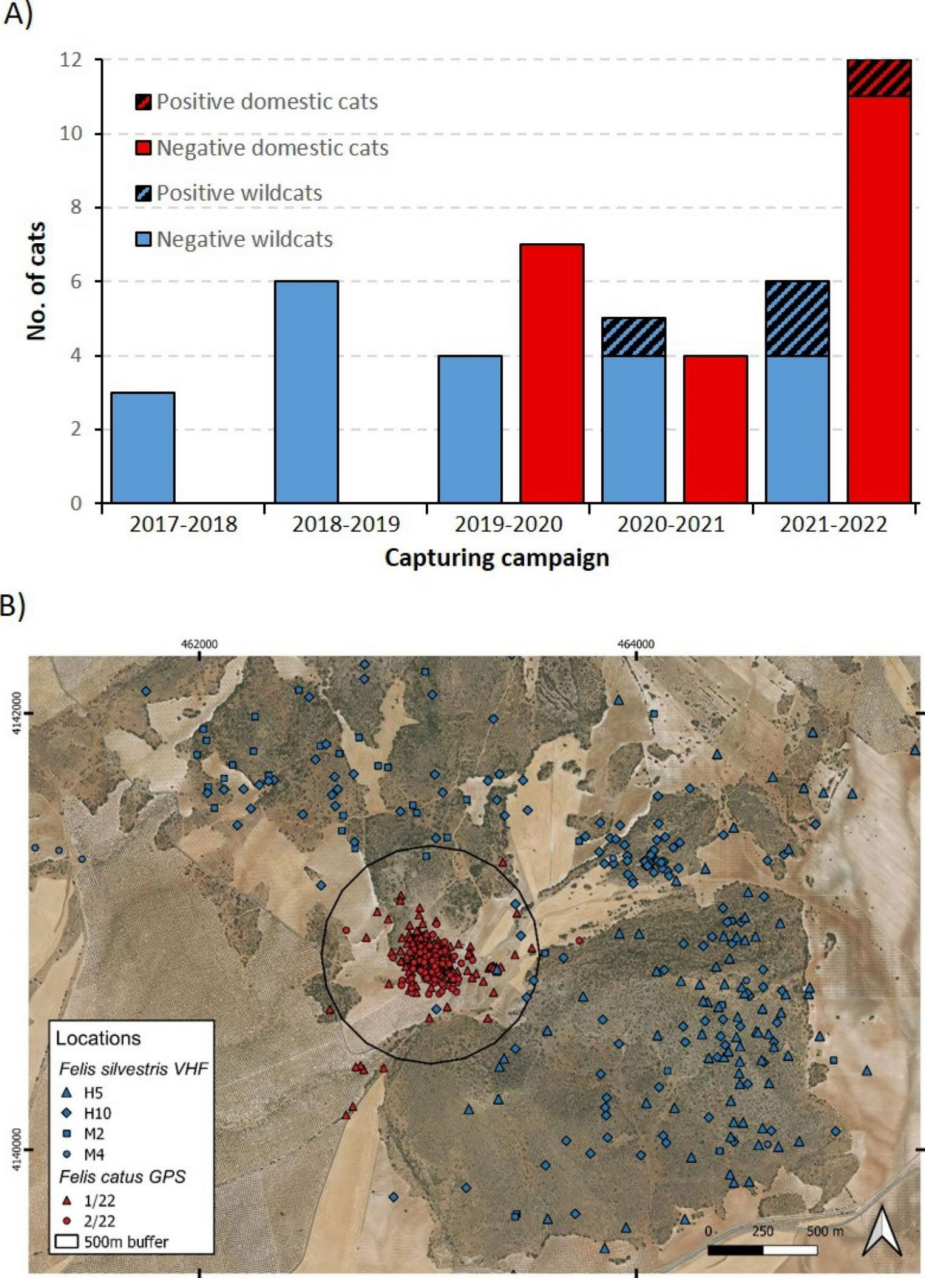
A) Positive and negative cats to the presence of *Thelazia callipaeda*, per capturing campaign. B) Locations of the radio-tagged wildcats around the locations of the GPS-tagged domestic cats. The female wildcat H5 and the male domestic cat 1/22 (plus the wildcats H3 and M13, which are not shown in the map; see main text for details) were positive to *T. callipaeda*

Out of the 17 wildcats checked, 6 were males (35%; 2 juveniles and 4 adults) and 11 were females (65%; 4 juveniles and 7 adults), while out of the 23 domestic cats, 13 were males (57%; 3 juveniles and 10 adults) and 10 were females (43%; 4 juveniles and 6 adults; Table S1). In total, three wildcats (17.6%) and one domestic cat (4.3%) were infected by ocular nematodes (Table 1; Fig. 1). The Chi^2^ test showed no inter-specific differences in prevalence (χ^2^ = 1.92, *P* = 0.16). In all positive cases, we observed eyeworms in both eyes. All nematodes collected were morphologically identified as *T. callipaeda*, and so-confirmed by molecular analyses. Nematodes were only detected since 2021, in autumn (November: *N* = 2 wildcats) and winter (February: *N* = 1 wildcat; March: *N* = 1 domestic cat; see Table 1; Fig. 2a). The two wildcats infected in November 2021 were previously checked (H3: November 2018 and October 2019; H5: November 2018; see Table S1), but no nematodes were detected by then (Fig. 2a).

**Table 1:** Information of the cases of the nematode *Thelazia callipaeda* detected in wildcats and domestic cats during our systematic surveys carried out in Sierra Arana (southeastern Spain) between 2017 and 2022

Date of sampling	Host species	Code	Sex	Age	Number of collected nematodes (males/females) [left/right eye]	Stage and clinical signs (Marino et al. 2021)
10/02/2021	*F. silvestris*	M13	male	juvenile	3 (0/3) [1/2]	Stage 3. Superficial corneal ulcer and uveitis
13/11/2021	*F. silvestris*	H5	female	adult	2 (1/1) [2/0]	Stage 2. Superficial corneal ulcer
20/11/2021	*F. silvestris*	H3	female	adult	2 (1/1) [2/0]	Stage 1. No clinical signs
19/03/2022	*F. catus*	1/22	male	adult	3 (1/2) [2/1]	Stage 1. No clinical signs

We obtained 1,280 locations for the radio-tagged wildcats (tracking period: 2017–2022) and 1,044 for the GPS-tagged domestic cats (tracking period: 2021–2022). Four wildcats were detected around the farm inhabited by the GPS-tagged domestic cats, resulting in seven locations (0.9% of the 763 locations of the four wildcats that moved around the farm) within a 500 m radius around the farm (see Fig. 2b). In addition, 0.8% of the locations of the two GPS-tagged domestic cats fell outside the 500 m buffer zone (Fig. 2b). One of these four wildcats (H5) and one of the farm’s domestic cats (1/22; Table 1; Fig. 2b) were infected by *T. callipaeda*. The home range of H5 partially overlapped with the home range of other infected wildcat (H3 in Table 1; Fig. 2b). The third infected wildcat (M13, not tagged) was captured 3.1 km outside of the home range of H5.

## Discussion

To our knowledge, this is the first report of *T. callipaeda* infecting wildcats in Spain. According to the recent spread of this nematode throughout Europe (Marino et al. 2020), it was a matter of time before detecting it in Spanish wildcats (Nájera et al. 2020). Some authors have suggested that the diagnosis rate is lower in felines compared to other mammals, because the former have less contact with the vector, their eyes are more difficult to inspect, they have cleaning habits that can remove secretions, and their lower body mass could be less attractive to the *Phortica* flies (Otranto et al. 2003b; Motta et al. 2014). However, given that *T. callipaeda* had been previously detected in domestic cats in Spain for over a decade (e.g., Miró et al. 2011), the lack of detection in wildcats could be mostly explained by the scarcity of systematic specific survey programs. Further research is needed to trace the colonization pathway in our study area.

We did not find differences in the prevalence of *T. callipaeda* between wildcats and domestic cats. This may be due to the fact that many domestic cats in the study area, especially farm cats, are widely exposed to the vector because they have an outdoor lifestyle and do not receive any sanitary management. In addition, farm cats strongly aggregate around farms (see Fig. 2b), which could facilitate the transmission of the parasite. Though the epidemiology of *T. callipaeda* in our study area is a dynamic process whose medium- to long-term fate is difficult to predict, we expect that prevalence values will increase in both cat species in the near future. Moreover, our results suggest that even relatively little spatial overlap between home ranges may pose an inter-specific transmission risk, especially considering that the vector is a fly. Also, in our study area, domestic cats are notably more abundant than wildcats (see the description of the study area above). Thus, the sanitary management of domestic cats, e.g., through regular veterinary examinations and preventive and curative anti-parasitic treatment, seems necessary to minimize the risk of threatened wildcats being infected by *T. callipaeda*. In turn, a lower prevalence of the nematode in the environment would also diminish the risk to human health (López-Medrano et al. 2015). Further eco-epidemiological long-term monitoring is needed to understand the dynamical relationship between the vector of this nematode and their hosts, including humans and other wild species in the study area, particularly mesocarnivores such as foxes, badgers, and stone martens (Otranto et al. 2009; Mihalca et al. 2016; Ionică et al. 2019), and two locally abundant lagomorphs, rabbit and hares (Otranto et al. 2009; Gama et al. 2016).

The eyeworms were found during the autumn and winter months (November, February, and March), when the average temperature was too low to support the circulation of its vector (Otranto et al. 2006). This suggests that *T. callipaeda* is endemic in the study area, remaining in its reservoirs during the coldest part of the year.

Our findings suggest the emergence of a new risk to the endangered Mediterranean populations of wildcats, as *T. callipaeda* could limit the fitness of infected animals. The ocular lesions can affect the correct vision (Diakou et al. 2015), making hunting more difficult, increasing the risk of being road-killed (an important mortality cause for European wildcats; Bastianelli et al. 2021), or producing critical bacterial infections (see Rolbiecki et al. 2021 for dogs). It is important to note that even apparently small threats could seriously hamper the viability of small wildcat populations, such as those in the Mediterranean region (Gil-Sánchez et al. 2020). Moreover, suitable habitats for Mediterranean wildcats are within the temperature preferences of *Phortica* flies, especially in a global warming scenario (Otranto et al. 2006; González et al. 2022). Thus, more studies are needed on the epidemiology of *T. callipaeda* and its effects on Mediterranean wildcat populations, particularly vulnerable to global warming (Lozano and Malo 2012).

### Electronic supplementary material

Below is the link to the electronic supplementary material.

Supplementary Material 1

## Data Availability

The datasets analyzed during the current study are available from the corresponding author on reasonable request.
