# Supplementary material for: Thelazia callipaeda as a potential new threat to european wildcats: insights from an eco-epidemiological study
Source: Vet Res Commun. 2023 Jan 17;47(4):2153–60. doi: 10.1007/s11259-023-10071-8 (PMC10697904; doi:10.1007/s11259-023-10071-8)
Supplement: Supplementary file 1 — Supplementary Material 1 [file 11259_2023_10071_MOESM1_ESM.docx]

Supporting Information for:

***Thelazia callipaeda* as a potential new threat to European wildcats: insights from an eco-epidemiological study**

Elena Bertos, Mariola Sánchez-Cerdá, Emilio Virgós, José M. Gil-Sánchez and Marcos Moleón

This material includes:

**Table S1.** Number of wildcats and domestic cats sampled in the study area, per capturing campaign.

**Table S1.** Number of wildcats and domestic cats sampled in the study area, per capturing campaign. Individuals with nematodes (*Thelazia callipaeda*) are indicated with an asterisk.

| **Species** | **Code** | **Sex** | **Age** | **2017-2018** | **2018-2019** | **2019-2020** | **2020-2021** | **2021-2022** |
| --- | --- | --- | --- | --- | --- | --- | --- | --- |
| *F. silvestris* | M1 | male | adult | X | - | - | - | - |
| *F. silvestris* | M2 | male | adult | - | X | - | - | X |
| *F. silvestris* | M4 | male | adult | - | X | X | X | - |
| *F. silvestris* | M9 | male | adult | - | X | - | - | - |
| *F. silvestris* | M13 | male | juvenile | - | - | - | X* | - |
| *F. silvestris* | M15 | male | juvenile | - | - | - | X | - |
| *F. silvestris* | H1 | female | adult | X | - | - | - | - |
| *F. silvestris* | H2 | female | adult | X | - | - | - | - |
| *F. silvestris* | H3 | female | adult | - | X | X | - | X* |
| *F. silvestris* | H4 | female | juvenile | - | X | X | - | - |
| *F. silvestris* | H5 | female | adult | - | X | - | - | X* |
| *F. silvestris* | H6 | female | juvenile | - | - | X | - | - |
| *F. silvestris* | H9 | female | juvenile | - | - | - | X | - |
| *F. silvestris* | H10 | female | adult | - | - | - | X | - |
| *F. silvestris* | H11 | female | adult | - | - | - | - | X |
| *F. silvestris* | H12 | female | juvenile | - | - | - | - | X |
| *F. silvestris* | H13 | female | adult | - | - | - | - | X |
| *F. catus* | 1/20 | female | adult | - | - | X | - | - |
| *F. catus* | 2/20 | male | adult | - | - | X | - | - |
| *F. catus* | 3/20 | male | adult | - | - | X | - | - |
| *F. catus* | 4/20 | female | adult | - | - | X | - | - |
| *F. catus* | 5/20 | female | juvenile | - | - | X | - | - |
| *F. catus* | 6/20 | male | juvenile | - | - | X | - | - |
| *F. catus* | 7/20 | male | adult | - | - | X | - | - |
| *F. catus* | 1/21 | female | adult | - | - | - | X | - |
| *F. catus* | 2/21 | male | adult | - | - | - | X | - |
| *F. catus* | 3/21 | female | juvenile | - | - | - | X | - |
| *F. catus* | 4/21 | female | juvenile | - | - | - | X | - |
| *F. catus* | 5/21 | male | adult | - | - | - | - | X |
| *F. catus* | 6/21 | male | juvenile | - | - | - | - | X |
| *F. catus* | 7/21 | male | juvenile | - | - | - | - | X |
| *F. catus* | 8/21 | female | adult | - | - | - | - | X |
| *F. catus* | 9/21 | male | adult | - | - | - | - | X |
| *F. catus* | 10/21 | female | adult | - | - | - | - | X |
| *F. catus* | 11/21 | male | adult | - | - | - | - | X |
| *F. catus* | 12/21 | female | adult | - | - | - | - | X |
| *F. catus* | 1/22 | male | adult | - | - | - | - | X* |
| *F. catus* | 2/22 | male | adult | - | - | - | - | X |
| *F. catus* | 3/22 | female | juvenile | - | - | - | - | X |
| *F. catus* | 4/22 | male | adult | - | - | - | - | X |
